# Miscellany

**Published:** 1888-09

**Authors:** 


### MISCELLANY.

THE NATIONAL FORMULARY.—A new "National Formulary" has been prepared by a committee of one from each State Pharmaceutical Association, appointed by the American Pharmaceutical Association in 1885. The purpose of the "Formulary" is to present an "authoritative standard" for the preparation of the floating formulas that have not heretofore been thought to possess sufficient merit to justify their recognition by the U. S. Pharmacopœia, and also of such new preparations as have acquired character and use since the last revision of the U. S. Pharmacopœia. *The Drugman* says: Every pharmacist is familiar with the trouble and annoyance which a want of uniformity in these migrating formulas occasions. Local variations causing slight difference in color, odor, or taste, have caused many a pharmacist to be suspected of having made a mistake. With a view of preventing as far as possible these annoyances, and for the more important purpose of establishing something like uniformity in the therapeutic strength and properties of these circulating formulas, this work was undertaken, and we presume it is fair to say that the book, whether good or bad, fairly represents the best American pharmaceutic talent at present. We have always believed that the work would retard rather than advance the principal purpose sought to be attained—uniformity. We have already a formulary prepared by a committee of physicians and pharmacists that has been universally received as authoritative, and no intelligent druggist considers it at all admissible to permit his officinal preparations to vary in strength or medicinal virtues from this recognized standard. It will certainly produce a great deal of confusion and mischief to have two standards, the pharmacist feeling authorized to follow his individual judgment in deciding which he shall obey in the preparation of medicinal compounds. The "National Formulary" will fill a want, and be very convenient until the next revision of the U. S. Pharmacopœia, when the trouble will begin. Many of the new formulas in the "National Formulary" will be adopted by the U. S. Pharmacopœia, with such modifications as the intervening experience may seem to render advisable. Some of these modifications will prove to be improvements, others will not, and where differences exist some pharmacists will prefer U. S. Pharmacopœia formulas and processes, and others the "National Formulary." We will thus have two standards, which is equivalent to no standard, and the object sought to be attained will be placed farther off. We can not have two standards. If they are identical, one answers every purpose; if they differ, the result will be damaging to every phase of medical progress. What we want is uniformity in officinal preparations—absolute uniformity as nearly as possible—and this can only be attained by following implicitly a single standard, and no departures from this guide should be considered proper or honorable except in isolated cases when proper explanations can be made. In the absence of uniformity the practitioner has no criterion by which to scientifically estimate the physiological and therapeutic effects and value of medicinal agents, and until this point shall have been attained medicine can never be a positive science. If the improved processes and floating formulas increase so fast that an official or semi-official collection is rendered necessary oftener than every ten years, let the U. S. Pharmacopœia be revised every five years. If none of the processes and formulas of the "National Formulary" could ever get in the U. S. Pharmacopœia, with modifications affecting their physical appearance or therapeutic strength and properties, there might be no objection to its existence on general principles; but this can not be prevented."

---

SULFONAL. — Regarding this hypnotic, discovered in 1886, Professor A. Kast, of Freiburg, says in the *Berliner Klinische Wochenschrift*, after trying it in sixty separate clinical cases, "Sulfonal deserves a prominent position among medicinal agents, not only as a hypnotic for producing temporary insensibility, but for its property of assisting the normal periodical want for sleep, and producing such where wanting; and furthermore because it produces no harmful effect on the elements of the blood; that it does not attack and impair the tissures of the alimentary channels; and that even in large doses it calls forth no dangerous disarrangement of the function of the heart or the vascular system."

---

Dr. Edward C. Huse, of Rockford, writes to say that in cases in which he has used salol (salicylate of phenol), in affections of the alimentary canal, as in cholera morbus and diarrhœa of children, the result has been so satisfactory that he regards it as a germicide of great value. On theoretical grounds he thinks it would be of much service in the treatment of cholera. He states that he was himself greatly benefited by taking it when suffering with inflammatory rheumatism, indicating usefulness in other conditions resulting from derangement of the digestive apparatus.

---

Messrs. Lehn & Fink, in "Notes on New Remedies," say: Gum Arabic, expensive and adulterated as it is now, can be advantageously replaced by casein in making emulsions. Cadet de Cassicourt was the first to direct attention to this substitute, and experiments have proved the value of his discovery. Emulsions prepared with casein remain permanent without separation of the oil, and have a pleasant taste, making the administering more palatable for the patient.

---

Topical Treatment of Varices.—Robert gives the following formula:

Chloride of sodium.......... 1 gramme.
Dissolve in
Distilled water................. q. s.
Shake and add
Lanolin................. 15 grammes.
Olive oil................. 5 "

For external use. Rub this over the region of the varicose veins three times a day.—*Revue de Therap. generale et Thermale.*

---

Diuretic Action of Beer.—R. Mori asserts that the diuretic action of beer corresponds only to that of a volume of liquid equal to one-third of the beer taken, and that more abundant diuresis is caused by wine. The diuretic action of beer and of alcoholic drinks should be attributed to the alcohol.—*Chemiker Zeitung*, 1888, xii, 32.

